# Factors Associated with Anti-Tuberculosis Medication Adverse Effects: A Case-Control Study in Lima, Peru

**DOI:** 10.1371/journal.pone.0027610

**Published:** 2011-11-16

**Authors:** Kocfa Chung-Delgado, Alejandro Revilla-Montag, Sonia Guillen-Bravo, Eduardo Velez-Segovia, Andrea Soria-Montoya, Alexandra Nuñez-Garbin, Wilmer Silva-Caso, Antonio Bernabe-Ortiz

**Affiliations:** 1 School of Medicine, Faculty of Health Sciences, Universidad Peruana de Ciencias Aplicadas, Lima, Peru; 2 Sociedad Científica de Estudiantes de Medicina, Universidad Peruana de Ciencias Aplicadas SOCIEMUPC, Lima, Peru; McGill University, Canada

## Abstract

**Background:**

Long-term exposure to anti-tuberculosis medication increases risk of adverse drug reactions and toxicity. The objective of this investigation was to determine factors associated with anti-tuberculosis adverse drug reactions in Lima, Peru, with special emphasis on MDR-TB medication, HIV infection, diabetes, age and tobacco use.

**Methodology and Results:**

A case-control study was performed using information from Peruvian TB Programme. A case was defined as having reported an anti-TB adverse drug reaction during 2005–2010 with appropriate notification on clinical records. Controls were defined as not having reported a side effect, receiving anti-TB therapy during the same time that the case had appeared. Crude, and age- and sex-adjusted models were calculated using odds ratios (OR) and 95% confidence intervals (95%CI). A multivariable model was created to look for independent factors associated with side effect from anti-TB therapy. A total of 720 patients (144 cases and 576 controls) were analyzed. In our multivariable model, age, especially those over 40 years (OR = 3.93; 95%CI: 1.65–9.35), overweight/obesity (OR = 2.13; 95%CI: 1.17–3.89), anemia (OR = 2.10; IC95%: 1.13–3.92), MDR-TB medication (OR = 11.1; 95%CI: 6.29–19.6), and smoking (OR = 2.00; 95%CI: 1.03–3.87) were independently associated with adverse drug reactions.

**Conclusions:**

Old age, anemia, MDR-TB medication, overweight/obesity status, and smoking history are independent risk factors associated with anti-tuberculosis adverse drug reactions. Patients with these risk factors should be monitored during the anti-TB therapy. A comprehensive clinical history and additional medical exams, including hematocrit and HIV-ELISA, might be useful to identify these patients.

## Introduction

Tuberculosis (TB) is considered a “global emergency” by the World Health Organization (WHO) [1]. Worldwide, about 9.4 million new cases are diagnosed annually and 1.7 million people die due to the infection. Moreover, over a third of the population has a latent infection with possibility of a posterior reactivation. Recently, 440,000 cases of multidrug resistant TB (MDR-TB) have been reported [1].

In Peru, around 35,000 cases of active TB are diagnosed every year, with morbidity and incidence rates of 118 and 103 per 100,000 people, respectively [2]. MDR-TB cases represent 5.3% of the total cases, and about 200 cases of extremely drug-resistant TB (XDR TB) have been reported [3], [4].

Usual treatment, according to the Peruvian National Health Strategy for Control and Prevention of Tuberculosis, is based upon DOTS strategy [2], [5]. The anti-TB therapy includes a long-time, wide spectrum of drugs depending on the characteristics of the TB infection: new cases, re-infection, relapses, failures, MDR-TB, or XDR-TB. Overall, this situation can predispose patients to develop adverse drug reactions due to the long-time exposition to the anti-TB drugs [6], [7].

Anti-TB drug side effects are an inherent risk for patients commencing any type of anti-TB therapy, especially the drug-resistant cases. The Peruvian Ministry of Health has notified a side-effects prevalence of 3.3% amongst treated patients [3]. The emergence of side effects may depend on patients' characteristics but also on concomitant events during therapy [8], which might determine adherence and, therefore, therapy success. Thus, an appropriate determination of risk factors associated with anti-TB medication adverse reaction is needed. The objective of this study was to assess factors associated with tuberculosis treatment side effects in Lima, Peru, with special emphasis on MDR-TB medication, HIV infection, diabetes, age, and tobacco use, adjusting for potential confounders.

## Methods

### Study Design, Setting and Participants

A retrospective, case-control study was performed using information from the National Health Strategy for Control and Prevention of Tuberculosis in Lima, Peru. This research used programmatic TB diagnosis, treatment monitoring and outcome data as recorded from the National Strategy for Prevention and Control of Tuberculosis at the San Juan de Miraflores-Villa Maria del Triunfo Health Network, periurban districts located at the south of Lima.

Those patients who had completed anti-TB treatment for pulmonary tuberculosis in any health facility of one of the districts aforementioned during 2005–2010 were considered for this investigation. Patients with XDR-TB were excluded from analysis due to their high probability of adverse effects and the individualization of treatment schemes. Patients with extra-pulmonary TB were also excluded from analysis because overall it is not possible to confirm the diagnosis (bacterial and histopathological confirmation), as well as the use of non-TB drugs such as corticoids during the active disease.

### Variable Definition

An adverse reaction was defined as having a harmful and non-intentioned effect due to anti-TB drug administration at an appropriate dose, being reported in clinical records with the respective notification documentation [2].

In the same way, according to clinical records and TB Programme information [3], the following data was extracted for analysis—all at the beginning of their respective anti-tuberculosis treatment: MDR-TB medication (yes/no), HIV infection (yes/no), diabetes (yes/no), and current smoking status (yes/no). Other variables included were: anemia (yes/no), defined using hemoglobin levels (<13.0 mg/dL in men and <12.0 mg/dL in women) [9]; current alcohol use (yes/no); and body mass index (BMI), in kg/m^2^, defined as normal (18.5≤BMI<25), overweight/obese (BMI≥25), and underweight (BMI<18.5) [10].

### Cases and Control Definition

A case was defined as having reported any TB-drug adverse effect (mild, moderate or severe) during 2005–2010 with the appropriate adverse effect notification and clinical record, both part of the TB Programme report. They must have included at least two more variables of interest, not including sex. A control was defined as those not having reported a side effect, receiving anti-TB therapy during the same time that the case had appeared. Thus, when a case was found, the next 4 controls sequentially commencing treatment at the same time were selected from the TB Programme records.

### Procedures and Data Collection

Routinely, during anti-TB therapy, patients are closely supervised until completion. When any adverse reaction emerges, health workers should immediately report it in clinical records using the appropriate TB Programme formats—as according to the Peruvian National Health Strategy for Prevention and Control of Tuberculosis and the Peruvian Ministry of Health [2]. Data was collected directly from the clinical records and TB Programme formats in the health facilities involved in the study. In the case of hemoglobin levels, data was obtained from the Nutritional Evaluation requested to all TB patients in accordance to the Peruvian Health Ministry Protocol. This information, however, was available only for patients who were explicitly dosed for hemoglobin levels based upon the nutritionist criteria (about 70% of the total) [2]. No personal identifiers were recorded to maintain anonymity and confidentiality of the patients.

### Sample Size and Statistical Analysis

Sample size was determined by including 144 cases, patients with anti-TB treatment side effects, and 576 controls (ratio 1∶4) during the period of the study, with a power of 80% to detect an odds ratio of 3.2 or more at the 5% level of statistical significance, assuming exposures of 3% or greater. Thus, a total of 720 patients were including in this study.

Data was analyzed using STATA version 11.0 for Windows (STATA Corporation, College Station, Texas, US). First, a brief description of demographic and clinical characteristics of the population was tabulated. Second, association between categorical variables and TB-drug adverse effects was assessed individually using Chi squared (χ^2^) and Fisher's exact test accordingly. Strength of the association was calculated using logistic regression analysis. Crude, and age- and sex-adjusted models were calculated using odds ratios (OR) and 95% confidence intervals (95%CI). Variables with p<0.15 in the bivariate model were used to create a final multivariable model using a stepwise forward selection method to look for independent factors associated with side effect due to anti-tuberculosis therapy.

### Ethics

This research proposal was approved by the School of Medicine, Faculty of Health Sciences, of the Universidad Peruana de Ciencias Aplicadas, Lima, Peru, as well as the Ethics Committee of the Epidemiology Division in the National Health Directorate II (DISA II), part of the Ministry of Health (Figure S1). No personal identifiers were recorded to maintain anonymity and confidentiality in this study. Informed consent was waived by IRB because of the use of routine and programmatic data of the National Health Strategy for Control and Prevention of Tuberculosis.

## Results

A total of 720 (144 cases and 576 controls, ratio 1∶4) were included in the study. Of them, 62.2% were males with an age mean of 31.8 (SD: 14.5; range: 3–84) years. See Table 1.

**Table 1:** Characteristics of the Population.

	n = 720	%
**Sex**		
Male	446	62.2%
Female	272	37.8%
**Age**		
Mean (SD†)	31.8 (±14.5)	
**Year of Diagnosis**		
2005	52	7.8%
2006	100	15.0%
2007	122	18.3%
2008	183	27.4%
2009	152	22.8%
2010	58	8.7%

† *SD: Standard deviation*.

Factors associated with anti-TB drug adverse effects after adjusting for age, sex and year of diagnosis were: alcohol use (p = 0.009), anemia (p = 0.001), overweight/obese condition (p = 0.03), HIV infection (p = 0.002), and MDR-TB infection (p<0.001). See Table 2.

**Table 2:** Factors Associated with TB-Treatment Adverse Effects.

Variable	Cases	Controls	p value	Crude Model	Adjusted Model*
	n (%)	n (%)		OR (95%CI)	OR (95%CI)
**Sex**					
Female	55 (38.2)	217 (37.7)	0.91	1	–
Male	89 (61.8)	359 (62.3)		0.98 (0.67–1.42)	–
**Age**					
<20 years	19 (13.2)	142 (24.7)	0.003	1	–
20–39 years	77 (53.5)	303 (52.6)		1.90 (1.11–3.26)	–
40+ years	48 (33.3)	131 (22.7)		2.74 (1.53–4.90)	–
**Alcohol Use**					
No	90 (64.3)	428 (75.5)	0.007	1	1
Yes	50 (35.7)	139 (24.5)		1.71 (1.15–2.54)	1.80 (1.16–2.80)
**Anemia**					
No	82 (69.5)	323 (81.4)	0.006	1	1
Yes	36 (30.5)	74 (18.6)		1.92 (1.20–3.05)	2.29 (1.37–3.83)
**Body Mass Index**					
Normal	69 (57.5)	319 (69.7)	0.02	1	1
BMI≥25 kg/m^2^	37 (30.8)	89 (19.4)		1.92 (1.21–3.05)	1.72 (1.04–2.84)
BMI<18.5 kg/m^2^	14 (11.7)	50 (10.9)		1.29 (0.68–2.47)	1.35 (0.68–2.68)
**Diabetes Mellitus** ††					
No	126 (92.7)	526 (96.7)	0.05	1	1
Yes	10 (7.4)	18 (3.3)		2.32 (1.05–5.15)	1.33 (0.54–3. 24)
**HIV Infection** ††					
No	124 (89.2)	545 (97.0)	<0.001	1	1
Yes	15 (10.8)	17 (3.0)		3.88 (1.89–7.98)	3.45 (1.61–7.45)
**MDR-TB**					
No	74 (51.4)	529 (91.8)	<0.001	1	1
Yes	70 (48.6)	47 (8.2)		10.7 (6.84–16.6)	10.3 (6.39–16.5)
**Tobacco use**					
No	90 (64.3)	428 (75.5)	0.102	1	1
Yes	50 (35.7)	139 (24.5)		1.50 (0.92–2.45)	1.65 (0.97–2.81)

*Adjusted for age, sex and year of diagnosis.

†† *Association evaluated by Fisher's Exact Test*.

*Data might not add up to totals due to missing values*.

When logistic regression model was used to assess independent factors, only anemia (p = 0.01), age (p = 0.008), body mass index (p = 0.03), MDR-TB (p<0.001), and tobacco use (p = 0.04) were associated with TB drugs adverse effects. See Table 3.

**Table 3:** Multivariable Model of Independent Factors Associated with TB-Treatment Adverse Effects.

Variables	Multivariable model
	OR (IC95%)
**Age**	
<20 years	1
20–39 years	2.61 (1.17–5.79)
40 or more years	3.93 (1.65–9.35)
**Anemia**	
No	1
Yes	2.10 (1.13–3.92)
**Body Mass Index**	
Normal	1
BMI≥25 kg/m^2^	2.13 (1.17–3.89)
BMI<18.5 kg/m^2^	0.87 (0.36–2.14)
**MDR-TB**	
No	1
Yes	11.1 (6.29–19.6)
**Tobacco Use**	
No	1
Yes	2.00 (1.03–3.87)

## Discussion

In this case-control study, age, anemia, drugs associated to MDR-TB treatment, overweight/obesity, and smoking were independently associated with adverse reactions from anti-tuberculosis treatment. On the other hand, although not significant in multivariable model, alcohol use, and HIV infection were only associated after adjusting for age, sex and year of diagnosis.

According to our findings, there is an increasing risk of TB-drug adverse events when age increases. In previous reports, the occurrence of any major side effect has been associated with age, especially amongst the elderly [7], [11]. The frequency of adverse reactions has shown to increase in a progressive and direct form in relationship to age [7], [11]. Overall, vulnerability to adverse reactions are more probable at older ages—especially at a hepatotoxic level—due to a significant reduction in clearance rate of metabolized drug agents by the cytochrome P450 enzyme, changes in the hepatic blood flow distribution, as well as other factors affecting liver function [12], [13].

Anemia, a blood marker commonly related to chronic diseases [14], was another factor found in our independent multivariable model as well as in our age and sex-adjusted model. Although data on hemoglobin levels is not routinely recommended in the laboratory examination according to the International Standard for Tuberculosis Care (ISTC) [15], such assessment is sometimes required in the Peruvian context due to the multilevel evaluation made to all TB patients including a complete nutritional evaluation and follow-up [2]. Some studies qualify anemia and malnutrition as risk factors for the development of anti-tuberculosis drug adverse reactions [16], [17]. Some additional nutrition factors have been associated such as mid-arm circumference and hypoalbuminaemia [18]. Thus, it is not surprising that anemia could be another potential predictor of adverse reactions. However, a direct causal relationship is far from being established. Some evidence suggests that anemia may not at all be a risk factor for the appearance of adverse effects, but rather the adverse reaction itself [12], [16], [19]. Isoniazid and rifampicin may directly cause hemolytic anemia, as can pyrazinamide cause sideroblastic anemia [15]. Others have suggested that anemia is seen as part of the clinical manifestation of tuberculosis and as a consequence of a chronic disease (chronic disease induced anemia) [14]. In general, tuberculosis patients have a higher predisposition to develop gastrointestinal absorption problems, consequently leading to anemia as a secondary side effect—yet not a risk factor itself [14]. In addition, a selection bias might have occurred due to the nutritionist selection of anemic cases. Thus, further studies are needed in order to confirm and clarify these findings.

On the other hand, a body mass index more than 25 kg/m^2^ (overweight and obesity) was also associated with adverse reactions. Limited information is available regarding this association, but a prior investigation reported that drug toxicity might result in obese patients receiving total body weight doses [20]. Another study has overall reported that obesity can have some effects on drug metabolism which could increase possibility of adverse events [21]. Of notice, a BMI<18.5 kg/m^2^, reported as a risk factor for active TB [22], [23], [24], was not associated with adverse reactions in our multivariable model as previously describe in other papers [13], [18].

Drugs associated with MDR-TB treatment is one of the most important factors associated with adverse reactions, increasing risk around 11 times compared to those who received first-line therapy. A prior study involving MDR-TB patients in Peru reported that 95% of treated patients had a type of adverse reaction to second-line TB drugs, with a proportion of 54% as toxic reactions [25]. Others studies have found high prevalences (more than 50%) of adverse drug reactions among MDR-TB patients [26], [27] compared to the expected prevalence of mild reactions in those using first-line therapy (around 5–20%) [19]. In general, drugs for treating MDR-TB strains have greater toxicity effects and involve a long-term exposure (18 to 24 months), all in great contrast with the treatment of a sensitive strain of TB [28].

Finally, smoking was the last independent factor associated with TB-drug adverse reactions. A previous case-control study found a statistically significant association between smoking and liver toxicity due to pyrazinamide [29]. However, literature regarding this finding is limited and further studies are needed to corroborate results.

Our study also found that other variables could be associated with adverse reactions from anti-TB drugs. Alcohol use can predispose and accelerate hepatotoxic effects caused especially by isoniazid [30], [31] due to enzyme induction changes [13]. Drug use can also be associated with hepatotoxicity. Overall, drugs have several effects which might affect the liver's enzyme induction as well as predisposing to hepatitis infection especially among intravenous drug users [32], [33]. Co-infection with hepatitis B or C has proven to be a high risk for adverse drug effects [33]. Unfortunately, our study did not assess hepatitis B or C infection as previously described. Surprisingly, HIV infection was not an independent factor associated with drug reactions. Previous authors have reported that side effects are higher in HIV-positive than in HIV-negative patients [7], [34], especially due to parallel treatment with highly-active antiretroviral therapy (HAART) [35], [36]. In our context, a strong association between HIV infection and MDR-TB (data not shown) might explain why HIV infection is not part of the final multivariable model. Surprisingly, diabetes—an expected risk factor [37]—was not a risk factor for adverse events in this study.

Strengths of this study include the analysis of a reasonable number of cases including only patients with adverse drug reactions systematically reported by the National Health Strategy for Control and Prevention of Tuberculosis. However, this study has several limitations. First, because of the retrospective nature of our study, it is not possible to demonstrate a causal relationship between the variables. It was not possible to determine the exact time of hemoglobin assessment in relation to the appearance of adverse effects. Moreover, some selection bias could have been introduced since subjective nutritionist criteria was used to dose for hemoglobin levels. Besides, missing data might have affected our results. Nevertheless, our findings are similar to previous reports. Thus, we believe that effect of missing value was not important in our analysis. Second, we could not adjust our results for other well-recognized confounders (education level or economic status). Further studies are needed to corroborate our findings. This study was not meant to assess the specific drug involved in the adverse reaction. Finally, we could not assess the drug involved in the reaction as previous reports have performed. However, our primary interest was to find risk factors associated with the occurrence of anti-TB adverse drug reactions.

In conclusion, older age, anemia, drugs associated with MDR-TB treatment, overweight/obesity status, and smoking history are in risk to develop anti-tuberculosis adverse drug reactions. Alcohol use, drug consumption and HIV infection were also associated in our age- and sex-adjusted models. Patients with these risk factors should be carefully monitored during the anti-tuberculosis drug treatment (DOTS strategy). A comprehensive clinical history (smoking and body mass index) and additional exams, including hemoglobin, hematocrit, HIV-ELISA, might be useful to identify these patients.

## Supporting Information

Figure S1 Ethics Approval from the Peruvian Ministry of Health.
(JPG)
