# Supplementary figures and images for: Factors Associated with Anti-Tuberculosis Medication Adverse Effects: A Case-Control Study in Lima, Peru
Source: PLoS One. 2011 Nov 16;6(11):e27610. doi: 10.1371/journal.pone.0027610 (PMC3217998; doi:10.1371/journal.pone.0027610)

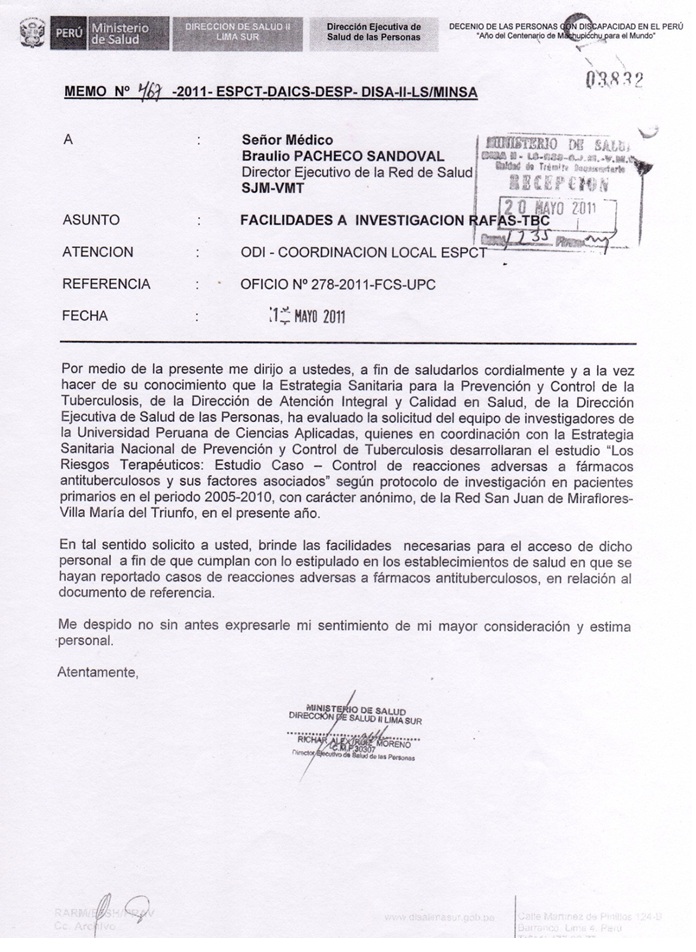

Supplement: Figure S1 — Ethics Approval from the Peruvian Ministry of Health. (JPG) [file pone.0027610.s001.jpg]
